# Assessing Overweight and Obesity Risk Among Korean Americans in California Using World Health Organization Body Mass Index Criteria for Asians

**Published:** 2006-06-15

**Authors:** Juhee Cho, Hee-Soon Juon

**Affiliations:** Johns Hopkins Bloomberg School of Public Health, Department of Health, Behavior, and Society; Johns Hopkins University Bloomberg School of Public Health, Department of Health, Behavior, and Society, Baltimore, Md

## Abstract

**Introduction:**

Although it is known that at comparable body mass index (BMI) levels Asian Americans have a higher prevalence of high blood pressure, heart disease, and type 2 diabetes than whites, little is known about the social, behavioral, and cultural factors associated with obesity risk in this population.

**Methods:**

A cross-sectional analysis of the 2003 California Health Interview Survey was performed to estimate overweight and obesity prevalence among Korean Americans using BMI criteria suggested by the World Health Organization for Asian populations worldwide. In addition, associations between demographics, social, behavioral, and cultural factors and the risk of being overweight and obese were examined.

**Results:**

Of 492 Korean American respondents, 38% were overweight and 8% were obese according to World Health Organization body mass index criteria for Asians. In a multivariate analysis, sex, marital status, poverty, and length of residence in the United States were associated with BMI. Men were more likely to be overweight or obese than women, and length of residence in the United States was strongly associated with higher body mass index.

**Conclusion:**

Like other ethnic groups, Korean Americans have a sociodemographic profile that is identified with an increased risk of becoming obese. Considering these factors in developing early diet and physical activity interventions could be an important opportunity to prevent weight gain and diminish disease caused by obesity. This study also suggests how meaningful BMI criteria tailored for Asian Americans could be used to more accurately measure risk of obesity within a heterogeneous population such as the U.S. population.

## Introduction

### Obesity epidemic in the United States

The United States is experiencing an epidemic of overweight and obesity. The prevalence of excess weight has been increasing rapidly across the country, and today almost 65% of the adult population is overweight or obese (1,2). From the period 1976–1980 to 1999–2000, the prevalence of overweight according to the Centers for Disease Control and Prevention (CDC) (body mass index [BMI] ≥25 kg/m^2^) has increased by approximately 40% (from 46% to 65%), and the prevalence of obesity (BMI ≥30 kg/m^2^) has risen by approximately 110% (from 15% to 31%) (2). Although some segments of the population are more likely to be overweight or obese than others, people of all ages, races, ethnicities, socioeconomic levels, and geographic areas in the United States are experiencing a substantial increase in weight (3,4).

As the prevalence of obesity has increased, concern about its health and economic consequences has also grown. Obesity has been linked to various chronic diseases, almost 300,000 deaths each year, and $117 billion in direct and indirect annual costs in the United States (1,4). It is associated with numerous illnesses, such as cardiovascular disease, type 2 diabetes, hypertension, stroke, dyslipidemia, osteoarthritis, and some cancers (5). In 2000, more than 17% of all deaths in the United States were attributable to being overweight or obese — a death rate surpassed only by those from tobacco use (6). According to CDC, poor diet and physical inactivity, both modifiable behaviors, contributed substantially to this obesity-related mortality (7).

### Obesity and Asian immigrants

Evidence suggests that racial and ethnic differences exist in the prevalence of obesity and the risk for obesity-related illnesses, particularly between whites and individuals who are black, Latino, or Asian, whether they are immigrants or were born in the United States (7). Many immigrants originate from countries where the prevalence of obesity is lower than it is in the United States (8), and they are found to be in better health than their counterparts who were born in the United States (9). However, these health advantages decrease with their duration of residence; the probability of becoming obese increases the longer the immigrants live in the United States (8,9). Some studies have found that the prevalence of obesity among adult immigrants living in the United States for at least 15 years approached that of adults born in the United States (3,7,8). For example, the percentage of Korean Americans who are obese (23%) is almost double the percentage of Koreans in Korea who are obese (12%) (10,11).

Although Asian American immigrants experience a lower prevalence of overweight and obesity than other ethnic groups (blacks, Latinos, and whites), they are at higher risk of developing chronic diseases than whites at the same BMI (12-15). The proportion of Asians with risk factors for type 2 diabetes and cardiovascular disease is substantial even below the BMI cutoff of 25 kg/m^2^, a BMI that typically indicates an increasing but acceptable risk for disease development for white or other ethnic groups (12-16). Asians usually have higher levels of abdominal fat at lower BMIs than whites (17,18) and have a higher percentage of body fat than whites of the same age, sex, and BMI (17-21). Although they have lower BMIs, Asian Americans in the United States are two times more likely than whites to have type 2 diabetes. BMI is one of the most important determinants of type 2 diabetes among some Korean, Chinese, and Japanese Americans (22). In addition, immigrants face more barriers to quality health care and are less likely to receive preventive health care than people born in the United States, making diagnosis of type 2 diabetes and other chronic disease even more difficult (23).

### California Korean Americans and their risk factors

The 2000 U.S. census indicated that Asian and Pacific Islanders were the second largest minority group in California, with a population of more than 3 million (24). Of the 14 Asian subgroups in California, Koreans are the fifth largest, contributing 12% of the Asian and Pacific Islanders population and 0.3% of the total U.S. population (24). Korean immigrants usually live in large cities with numerous other Korean immigrants (24,25). At least 50% of all Korean Americans reside in California, New York, and New Jersey, and the number of Korean Americans in California increased by 133% in the last decade (24). Even though their population is growing substantially in the United States, few studies or interventions about Korean Americans' health have been conducted (10,11,25).

Although Korean Americans generally have higher levels of education than other Asian subgroups and other ethnic groups in the United States, more Korean Americans reported being in fair or poor health than other subgroups of Asian and Pacific Islanders (26). Compared with Koreans in Korea, Korean Americans experience a higher prevalence of overweight and obesity and report more overweight- and obesity-related illnesses such as high blood pressure, type 2 diabetes, and heart disease (9-11). Korean Americans also have behavioral risk factors such as lack of exercise, inadequate routine physical examinations, inadequate numbers of screenings (e.g., mammograms, colorectal cancer screenings), smoking, and lack of health care access, all of which can increase risk of obesity (10,11,26-32).  

Obesity is a physically and financially costly disease, not only for the affected individuals and their families but also for the health care system and the entire U.S. population (1-4). Despite the increasing prevalence of obesity and the knowledge of various obesity-related risk factors, little attention has been paid to the prevalence of obesity and the related risk factors of Korean Americans specifically. This study examined the prevalence of overweight and obesity among Korean Americans in California and explored the relationship between social, behavioral, and cultural factors and overweight and obesity.

### The 2003 California Health Interview Survey

The 2003 California Health Interview Survey (CHIS 2003) is the largest health survey that has ever been conducted in California or any other state (33). The CHIS 2003 collected information by telephone interview from 42,000 randomly selected households; 54,580 people were interviewed (,42,818 adults aged 18 years or older, 4010 adolescents aged 12–17 years, and 8526 children aged 11 years or younger) (34). The CHIS sample was designed to produce reliable estimates for the entire state, including for medium and small counties, and provide estimates for California's overall population and its larger racial and ethnic groups as well as for several other ethnic groups (34).

CHIS 2003 is a valuable data source that can be used to examine the health needs of the heterogeneous Asian population. In addition to English and Spanish, the survey was administered in Cantonese, Mandarin, Korean, Vietnamese, and Khmer (33). To increase the precision of estimates for Koreans and Vietnamese, these two groups were oversampled by using a combination of geographic targeting and a surname-listed sample (34).

### World Health Organization (WHO) overweight and obesity BMI criteria

The purpose of a BMI cutoff point is to identify the proportion of people within each population with a high risk of a health condition that warrants a public health or clinical intervention (14). After a WHO expert consultation on appropriate BMIs for Asians, WHO reported that the current BMI cutoff points do not adequately reflect risks related to overweight and obesity in many Asian populations (14,16). Asian populations typically have a different percentage of body fat related to BMI and have a high risk for type 2 diabetes and cardiovascular disease at BMIs that are lower than the existing BMI cutoff point of 25 kg/m^2^ (14,16).

According to the WHO consultation, the additional BMI cutoff points for Asian populations are from 23 to 27.4 kg/m^2^ (overweight and at* increased risk *for BMI-associated disease) and 27.5 kg/m^2^ or higher (obese and at *high risk* for BMI-associated disease) (13,14,16).

### Factors affecting BMI

Demographics (e.g., age, sex, marital status) (35-37) and socioeconomic status (SES) (35,37,38), health behaviors (drinking alcohol, smoking) (36,38), and access to health care (37,39) are factors known to affect BMI. Specifically, SES indicators such as education, federal poverty level, and employment status have been shown to be associated with excess weight and obesity within various ethnic groups (7,35,36,38). Researchers have also found that long working hours increase feelings of stress and promote unhealthy lifestyle changes such as smoking, alcohol abuse, lack of physical activity, sleeplessness, poor eating habits, and fewer medical examinations (40,41). We hypothesized that the number of hours worked per week would be associated with higher BMI and that people who worked more than 40 hours per week would report unhealthier lifestyle behaviors and be more likely to be overweight or obese than people who worked 40 or fewer hours per week.

For immigrants, acculturation to U.S. norms can lead to the adoption of a more sedentary lifestyle (westernized lifestyle), which is one of the most important factors associated with the increasing prevalence of excess weight and obesity (7,8,10,11,42). The prevalence of becoming overweight and obese increases the longer immigrants live in the United States (7-11,42).

## Methods

Korean Americans have similar physical differences and risk factors as other Asian populations, so this study used the suggested WHO BMI cutoff points for Asians to assess several variables. Respondents whose BMI was 23 kg/m^2^ or higher were categorized as overweight and obese, and respondents whose BMI was less than 23 kg/m^2^ were categorized as normal weight.

Demographic variables included age (18–49 years, 50–81 years), sex, and marital status (married; separated, divorced, or widowed; never married). Education attainment was categorized into two groups (some college or more, high school graduate or less), and federal poverty level was categorized into three groups (≥300%, 100%–299%, ≤99%).

Working status was first categorized into two groups (employed and unemployed), and the employed group was divided into two groups (people who work 40 hours per week or less and people who work more than 40 hours per week). As proxy measures of acculturation, length of residence in the United States and spoken English proficiency were analyzed. However, because length of residence and spoken English proficiency were highly associated (χ^2^= 42.56,  *P* < .001) (data not shown), only length of residence was included in the final model.

Smoking status (current smoker or nonsmoker) and drinking habits (binge drinker or nonbinge drinker) were analyzed as health behavior indicators. People who reported on the survey that they smoked were classified as current smokers. Those who reported having quit smoking or never smoked regularly were classified as nonsmokers. Binge drinkers were classified as respondents who reported having had five or more drinks on one occasion in the previous month. Access to health care was measured by health insurance coverage (insured or not insured).

### Data analysis

To examine the risk of overweight and obesity, logistic regression was used to assess various levels of predictors. Bivariate analyses were performed to determine which independent variables would distinguish people with higher BMIs. Interactions between variables were tested based on the literature and behavioral plausibility. Finally, multivariate logistic regression analyses were performed to identify the most important predictors of having higher BMI while controlling for sociodemographic characteristics (age, sex, marital status, education level, employment, poverty level), acculturation (length of residence in the United States), health behaviors (drinking and smoking habits), and access to health care (health insurance coverage).

Descriptive and logistic regression analyses were weighted using SUDAAN (Research Triangle Institute, Research Triangle Park, NC) to account for the sample design (43). Estimates for the standard errors were computed using the jackknife method (43).

## Results

### Characteristics of Korean American adults in California

Table 1 shows characteristics of Korean American adults in California. The 492 eligible Korean American respondents represented an estimated 330,000 Korean American adults in California. Approximately 58% were women, and approximately 42% were men. The mean age of all respondents was 45.8 years, ranging from 18 to 81 years; 31% were aged 50 years or older. About 64% were married, and about 26% had never been married. About 60% of Korean Americans had lived in the United States 15 years or more or been born in the United States, and approximately 15% had lived in the United States fewer than 5 years.

Korean Americans were highly educated: 54% had a college education or more, 14% had some college education, and 32% had a high school or less than a high school education. Among Korean Americans, 54% were at or above the 300% federal poverty level, and 12% were below the 100% federal poverty level. Approximately 56% of Korean Americans were employed, and among them about 68% were working more than 40 hours per week. Approximately 13% said that they were in excellent health, and about 6% reported that their health was poor. About 20% were current smokers, and about 18% were binge drinkers. Approximately 30% did not have health insurance.

### BMI and sex differences

Table 2 shows sex differences in BMI. Approximately 38% of Korean Americans were overweight, and 8% were obese. This rate of overweight and obesity is higher than that of other ethnic groups and whites. We found that men were more likely to be overweight and obese than women (χ^2^ = 19.56, *P* < .001).

### BMI and social, behavioral, and cultural factors

Table 3 shows the results of the bivariate and the multiple regression analyses. In bivariate analyses, sex, number of working hours, length of residence in the United States, and health insurance coverage were associated with the likelihood of being overweight or obese. None of the health behaviors, such as smoking and drinking status, were associated with the likelihood of being overweight or obese. Bivariate analysis showed two statistically significant interactions: between poverty level and number of hours worked and between sex and marital status (data not shown). However, these two interactions were not significant within the multivariate analysis; thus, they were not assessed in the multivariate analyses.

A multivariate logistic regression model was created after the bivariate analyses. Sex, marital status, poverty, and years of residence in the United States were associated with the likelihood of being overweight or obese. Men were more likely to be overweight and obese (BMI ≥23) than women (OR, 2.89; 95% confidence interval [CI], 1.57–5.30). In bivariate analyses, marital status was not significantly associated with the likelihood of being overweight or obese; however, it was significantly associated in the multivariate analysis. People who had never married had a statistically significant lower risk of being overweight and obese (OR, 0.50; 95% CI, 0.25–0.98) than people who were married.

Examining the relationship between SES and BMI showed that BMI did not differ by poverty level in bivariate analysis, but in the multivariate analysis, people who were below the 100% federal poverty level were approximately 2.8 times more likely to be overweight or obese (OR, 2.77; 95% CI, 1.19–6.49) than people who were at or above the 300% federal poverty level.

A higher level of acculturation was associated with a higher BMI (Table 3). Korean Americans who had lived in the United States for 15 years or more or were born in the United States had more than twice the likelihood of being overweight and obese (OR, 2.45; 95% CI, 1.01–6.04) than people who had lived in the United States fewer than 5 years.

## Discussion

This study showed that approximately 50% of Korean Americans in California were overweight or obese based on WHO BMI criteria for Asians. The findings indicate that Korean Americans are at risk for obesity and obesity-related chronic diseases, although they have been previously categorized as healthy and generally not at risk (10,12). This study also suggests how different BMI criteria (especially for Asian Americans) could be used to measure risk of obesity within heterogeneous populations in the United States. It alerts us to the important public health data that could be missed when the same BMI criteria are used for all ethnic and racial subgroups in the U.S. population.

Our study showed that Korean American men were more likely to be overweight and obese than Korean American women. This is somewhat inconsistent with previous studies; overall, men had higher BMI scores than women, but there was some difference between overweight and obesity (35). Although the rate of overweight was higher among men, the rate of obesity was higher among women (35). Some researchers have said that differences in SES factors such as employment and income might differently affect body weight among men and women (38). However, we found no differences when comparing SES and BMI scores for Korean American men and women in this study.  

In our study, acculturation was associated with higher BMI among Korean Americans. Consistent with the findings of previous studies (7-11,37), Korean Americans with longer residence in the United States and who were born in the United States had a higher risk of overweight and obesity than more recent U.S. immigrants. Trends in obesity among Korean Americans may reflect acculturation and adoption of an American lifestyle, which includes more sedentary behavior and consumption of high-fat, high-sugar food. The obesity trend may also be a response to the physical environment of the United States, which has highly available high-fat and calorie-dense foods, higher reliance on private cars rather than public transportation, and greater participation in physically inactive types of entertainment such as watching television or movies.

Marital status was also an important factor affecting BMI among the population in our study. People who had never been married had a lower risk of being overweight or obese than people who were married. Sex and marital status had some effect, although it was not statistically significant in a multivariate model. Korean American men who had never been married had the lowest BMI scores, whereas Korean American women who were divorced, widowed, or separated had the highest BMI scores. This is consistent with previous findings that being married is positively associated with BMI scores in men but not women (44). In a future study, it would be interesting to examine other environmental and behavioral factors related to marital status and higher BMI, such as number of children, family activities, and household income, to determine whether they are associated with BMI scores. 

We found that poverty level was associated with BMI among Korean Americans. Lower-income Korean Americans were at a higher risk of being overweight or obese than higher-income Korean Americans. Previous studies of obesity and poverty in other populations have reported similar findings (35,36,38). The association between obesity and poverty may be explained by the observation that people with lower incomes are more likely to eat inexpensive, energy-dense foods (i.e., high sugar and high fat) (45). Although education is often considered a confounder of obesity and poverty (35,37), it was not associated with poverty level in this study. In addition, in previous studies, education has not been found to be an important factor for determining preventive health care and health behaviors among Korean Americans (29-31). Additional investigations are needed to examine the association between poverty level and increased risk of overweight and obesity among Korean Americans. 

Although no statistically significant relationship was found between number of working hours and BMI, the related findings are interesting. Approximately 70% of Korean Americans who were employed worked more than 40 hours per week. This group was at a higher risk of being overweight and obese than people who were not employed. Other researchers have found that long working hours increase feelings of stress and result in unhealthy lifestyles that include lack of physical activity and unhealthy eating habits (40,41). This might explain the results of our study, which found that people who worked more than 40 hours per week had an increased risk of being overweight and obese. People who work more hours may eat more quickly, eat irregularly, skip meals, or eat a lot at one time because of their busy schedules. It may be helpful to examine the relationships between the number of hours worked, other employment characteristics, and BMI. Many Asian immigrants are self-employed or work with family members (9,25), so it would be helpful to investigate whether people who are self-employed or involved with a family business are more likely to work more hours and whether they have a higher risk of being overweight or obese than those who work at a private company or in the government.

We found no relationship between healthy behaviors (not smoking, drinking alcohol in moderation or not at all) and BMI in this study. We were unable to determine which health behaviors affect each other within this population. However, it is important to consider health behaviors when assessing risk of overweight and obesity because people who practice one type of unhealthy behavior may also practice another, such as binge drinking, smoking, overeating, or lack of exercise (36,46). Based on the current study results, Korean Americans have relatively higher education levels than the average person in the United States: 54% of Korean Americans had more than 4 years of college education, compared with 24% of the entire U.S. population (24). However, Korean Americans have reported more unhealthy behaviors (e.g., higher rates of cigarette smoking) as well as fewer preventive health behaviors (e.g., lower rates of receiving mammograms and Papanicolaou [Pap] tests among women) (27-31). They also have less access to health care services than other ethnic groups in the United States (32). It could be helpful to know which social and environmental factors contribute to Korean Americans' engagement in behaviors that increase obesity risk, particularly considering their high level of education.

This study has two important limitations. First, data for BMI scores were based on self-report rather than on an objective measure of height and weight. Self-report can lead to recall bias. Although the interviews were conducted in Korean, cultural and linguistic differences may have also contributed to differential reporting of height and weight. Second, although the majority of Korean Americans live in California, CHIS data do show regional variations in BMI within the state (27). It is also unclear whether these findings are generalizable to Korean Americans living in other regions of the United States.

The prevalence and risk of overweight and obesity among Korean Americans is of concern given the rapid growth of the Korean American population in the United States and the adverse obesity-related health outcomes. Unfortunately, less attention and fewer interventions related to obesity have been directed toward Korean Americans and other Asian Americans, likely because most health surveys and research conducted in the United States have used the normal BMI criteria for classifying people as overweight (BMI ≥25 kg/m^2^) or obese (BMI ≥30 kg/m^2^). As the results of this study and other national studies show, comparably fewer Asian Americans are overweight or obese with a BMI cutoff point of 25; however, Asian Americans have a similar prevalence of type 2 diabetes, heart disease, and other chronic conditions that are primarily caused by obesity (12-16,22). Researchers need to replicate the current study findings using a larger sample that includes more Asian American subgroups.

In summary, using the suggested WHO BMI cutoff points for overweight and obesity in Asian populations, we found that Korean American adults in California seem to have a prevalence of obesity similar to that of white adults. The prevalence of obesity among Korean Americans increases as the duration of residence increases, as their marital status changes, and as their income levels decrease. Korean American men in the study experienced the greatest effects from these variables. Use of these factors to customize early interventions for diet and physical activity may be an important opportunity to decrease the prevalence of obesity and related disease among Korean Americans.

## Figures and Tables

**Table 1:** Characteristics of Korean American Adults in California, 2003 California Health Interview Survey (N = 492)

**Characteristics**	**% (SE)^a^**
**Demographics**	
Age, y (mean = 45.8)	
18-49	68.6 (1.98)
50-81	31.4 (1.98)
Sex	
Female	58.1 (2.36)
Male	41.9 (2.36)
Marital status	
Married	64.4 (3.35)
Divorced, widowed, or separated	9.9 (1.64)
Never married	25.7 (3.05)
**Acculturation**	
Length of residence in the United States, y	
<5	15.1 (2.11)
5-9	11.7 (1.93)
10-14	13.6 (2.58)
≥15 or born in United States	59.5 (2.83)
English proficiency	
Not at all, not well	45.7 (2.90)
Well	28.3 (2.62)
Very well, native	26.0 (3.29)
**Socioeconomic status**	
Highest education level	
≥4 y college	54.0 (3.14)
Some college	14.2 (2.40)
High school graduate or less	31.8 (2.79)
Federal poverty level, %	
≥300	53.9 (2.94)
200-299	14.9 (2.08)
100-199	18.8 (2.16)
≤99	12.4 (1.73)
Working hours per week	
0 (not working, unemployed)	43.9 (3.02)
≤40	18.1 (2.88)
>40	38.0 (2.97)
**General health condition**	
Excellent	13.3 (2.10)
Very good	25.6 (3.13)
Good	36.5 (3.01)
Fair	18.6 (2.21)
Poor	6.0 (1.16)
**Health behaviors**	
Smoking status^b^	
Nonsmoker	79.9 (2.35)
Smoker	20.1 (2.35)
Drinking status^c^	
Nonbinge	82.6 (2.67)
Binge	17.5 (2.67)
**Healtd insurance status**	
Insured	69.9 (2.91)
Uninsured	30.1 (2.91)

a All SEs were adjusted for design effect with SUDAAN (Research Triangle Institute, Research Triangle Park, NC).

b People who reported on the survey that they smoked were classified as current smokers. Those who reported having quit smoking or never smoked regularly were classified as nonsmokers.

c Binge drinkers were classified as respondents who reported having had five or more drinks on one occasion in the previous month.

**Table 2:** Body Mass Index (BMI) of Korean American Adults in California Using World Health Organization Criteria for Asian Adults, 2003 California Health Interview Survey (N = 492)

**BMI, kg/m^2^**	**Women, % (SE)^a^**	**Men, % (SE)^a^**	**Total, % (SE)^a^**	**χ^2^_df_**	***P *Value**
<23	64.09 (3.68)	40.30 (4.47)	54.11 (2.81)	χ^2^_2_ = 19.56	<.001
23-27.4 (overweight)	30.69 (3.82)	49.03 (4.99)	38.38 (2.95)		
≥27.5 (obese)	5.22 (1.31)	10.67 (2.82)	7.51 (1.36)		

a All SEs were adjusted for design effect with SUDAAN (Research Triangle Institute, Research Triangle Park, NC).

**Table 3:** Social and Behavioral Characteristics and Likelihood of Being Overweight or Obese Among Korean Americans in California (N = 492), 2003 California Health Interview Survey

**Variable**	**Likelihood of Being Overweight (BMI >23 kg/m^2^) or Obese (BMI ≥27.5 kg/m^2^)**	

	**Unadjusted Odds Ratio (95% CI^a^)**	**Adjusted Odds Ratio (95% CI^a^)**
**Age, y**		
18-49	Ref	Ref
50-81	1.20 (0.75-1.93)	1.02 (0.49-2.10)
**Sex**		
Female	Ref	Ref
Male	2.64 (1.63-4.29)	2.89 (1.57-5.30)
**Marital status**		
Married	Ref	Ref
Divorced, widowed, or separated	1.25 (0.65-2.43)	1.36 (0.60-3.04)
Never married	0.85 (0.43-1.67)	0.50 (0.25-0.98)
**Education**		
Some college or more	Ref	Ref
High school graduate or less	0.67 (0.39-1.14)	0.67 (0.34-1.35)
**Poverty, %**		
≥300	Ref	Ref
100-299	1 (0.59-1.70)	1.09 (0.59-2.04)
≤99	1.58 (0.78-3.20)	2.77 (1.19-6.49)
**Working hours per week**		
0 (not working, unemployed)	Ref	Ref
≤40	1.46 (0.59-3.62)	1.33 (0.48-3.69)
>40	1.61 (1.04-2.51)	1.15 (0.67-1.97)
**Length of residence in United States, y**		
<5	Ref	Ref
5-9	2.22 (0.82-5.99)	2.53 (0.77-8.24)
10-14	2.85 (0.95-8.51)	3.14 (0.85-11.62)
≥15 or born in United States	2.19 (1.13-4.25)	2.45 (1.01-6.04)
**Smoking status^b^**		
Nonsmoker	Ref	Ref
Smoker	1.22 (0.64-2.31)	0.89 (0.40-1.98)
**Drinking status^c^**		
Nonbinge	Ref	Ref
Binge	2.20 (0.94-5.17)	2.18 (0.82-5.78)
**Health insurance status**		
Insured	Ref	Ref
Uninsured	0.57 (0.33-0.99)	0.54 (0.27-1.06)

BMI indicates body mass index; CI, confidence interval; Ref, reference group.

a 95% CIs were computed based on weighting provided in the California Health Interview Survey.

b People who reported on the survey that they smoked were classified as current smokers. Those who reported having quit smoking or never smoked regularly were classified as nonsmokers.

c Binge drinkers were classified as respondents who reported having had five or more drinks on one occasion in the previous month.
